# Supplementary material for: Fossil evidence for vampire squid inhabiting oxygen-depleted ocean zones since at least the Oligocene
Source: Commun Biol. 2021 Feb 18;4:216. doi: 10.1038/s42003-021-01714-0 (PMC7893013; doi:10.1038/s42003-021-01714-0)
Supplement: Supplementary file 2 — Supplementary Information [file 42003_2021_1714_MOESM2_ESM.pdf]

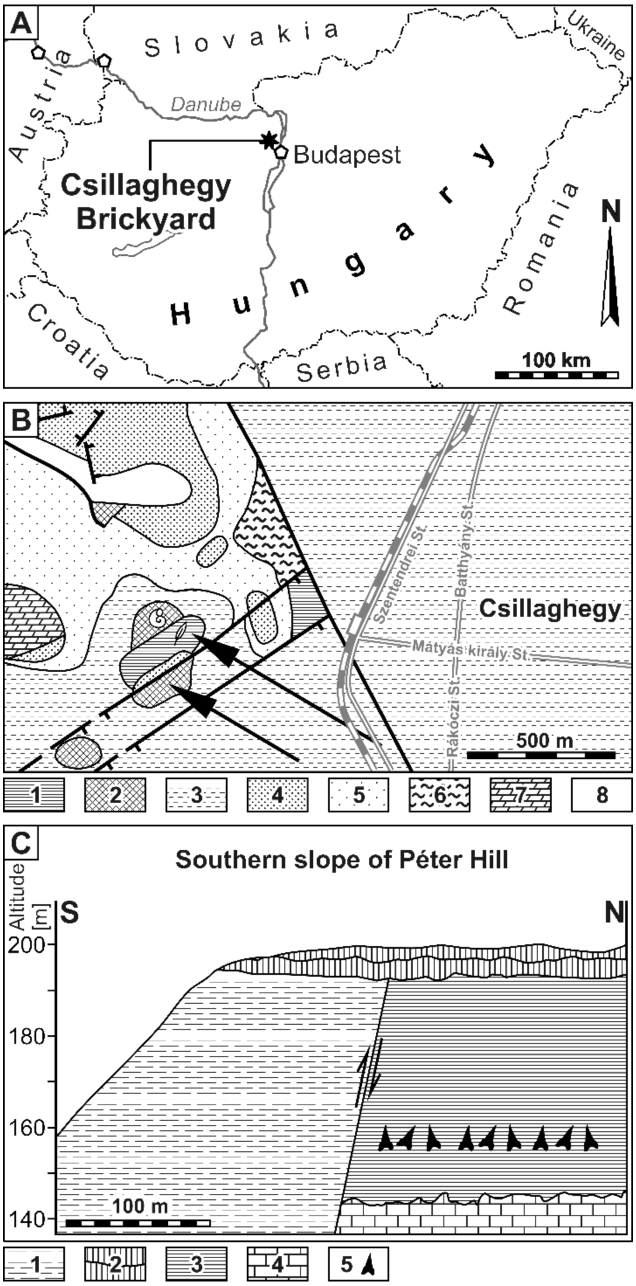

Supplementary Fig. 1. **a** Geographical position of the once existing Csillag-hegy brickyard (asterisk). **b** The surface outcrops of Oligocene formations surrounded by Eocene and Triassic rocks in the vicinity of the settlement Csillaghegy. The shorter and longer arrows mark the former outcrop of the the Kiscell Clay Fm. and Tard Clay Fm. (Csillag-hegy brickyard). 1) Oligocene Tard Clay Fm. (surface). 2) Oligocene Kiscell Clay Fm. (surface). 3) Oligocene Kiscell Clay Fm. (covered). 4) Eocene conglomerate, limestone with *Nummulites* or *Discocylina*, bryozoan marl (surface). 5) Eocene conglomerate, limestone with *Nummulites* or *Discocylina*, bryozoan marl (covered). 6) Eocene Buda Marl Fm. 7) Triassic grey dolomite (Hauptdolomite). 8) Triassic Diplopora Dolomite. **c** Geological profile of the former Csillag-hegy brickyard after[79]. The exact level of the *Necroteuthis* specimens within the Tard Clay Fm. is unknown. 1) Kiscell Clay Fm., 2) travertine, 3) Tard Clay Fm., 4) Upper Eocene carbonates, 5) level with abundant shark teeth in the lower part of the Tard Clay Fm.

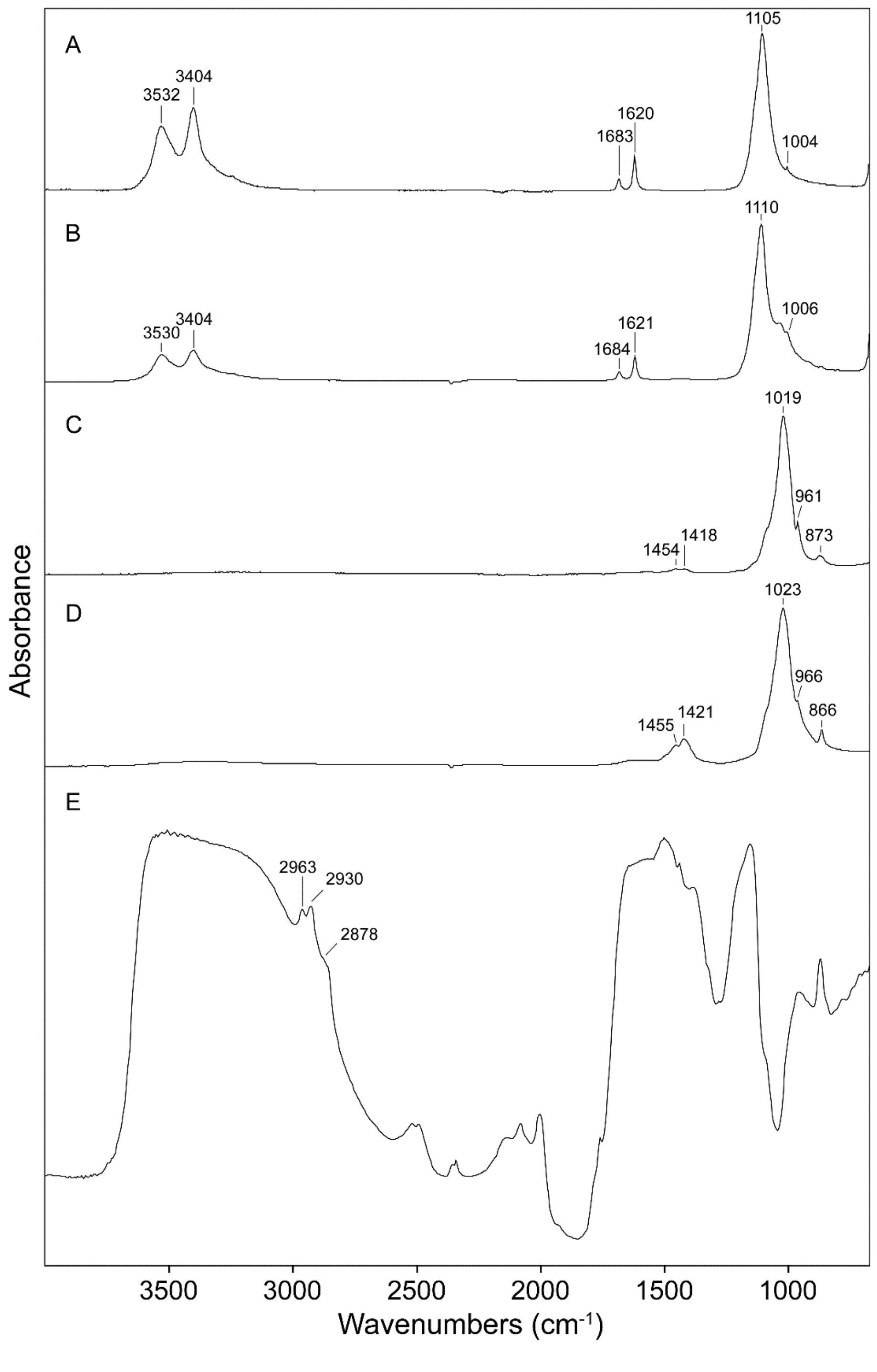

Supplementary Fig. 2. Recorded infrared spectra and reference spectra (Fourier-transform Infrared spectroscopy - FTIR). **a** reference spectrum of gypsum RRUFF R040029-1, **b** recorded spectrum of gypsum, **c** reference spectrum of (hydroxyl)apatite R050512-1, **d** recorded spectrum of (hydroxyl)apatite, **e** recorded spectrum of a mixture of fossilized/altered organic matter.

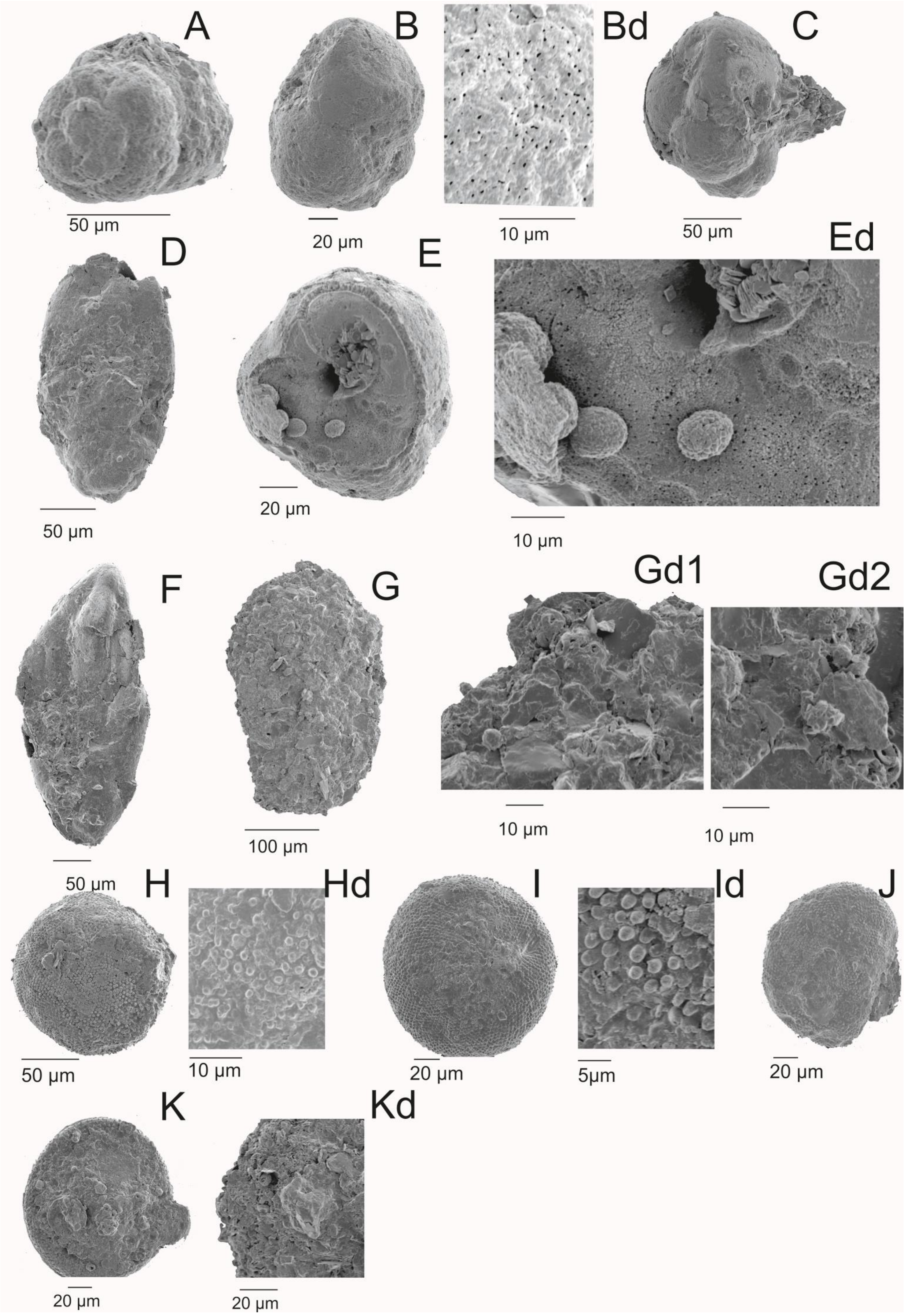

Supplementary Fig. 3. a-e *Caucasina* sp. cf. *Caucasina schischkinskayae* **bd** – detail of densely porous test. **d** Collapsed younger chambers in larger adult specimen. **e** Bacterial activity signs, inner septum with framboidal pyrite pseudomorphoses after bacteria (**ed**). **f** *Fursenkoina acuta* (compressed). **g** ?*Miliammina* sp. **gd1**, **gd2** Nannofossils incorporated into the test wall. **h-k** *Tasmanites* sp. **hd**, **id** Details of the external wall structure. **j** Inner cavity. **k**, **kd** Calcareous nannoplankton crust (integument) on the *Tasmanites* sp. wall.

Supplementary Micro CT imaging:

The micro-computed tomography images generated during and/or analysed during the current study are available in the figshare repository: https://doi.org/10.6084/m9.figshare.13526024[90]. The *Necroteuthis* holotype which is the subject of the imaging is housed in the Hungarian Natural History Museum under item: No. M59/4672.

Supplementary Tables 1-3.

	Sample 1	Sample 2	Sample 3	Sample 4	Sample 5	
Coccolithus pelagicus (Wallich 1877) Schiller, 1930	11.18	36.07	56.99	43.00	32.73	NP2-Recent
Coronocyclus nitescens (Kamptner, 1963) Bramlette and Wilcoxon, 19	0.74	0.00	0.00	2.00	0.00	NP15-NN6
Coronocyclus nitescens (Kamptner, 1963) Bramlette and Wilcoxon, 1967	0.00	0.00	1.08	0.00	0.00	NP15-NN6
Cyclicargolithus floridanus (Roth & Hay, *in* Hay *et al.*, 1967) Bukry, 1971	2.94	1.64	0.00	1.00	0.91	NP15-NN7
Discoaster nodifer (Bramlette & Riedel, 1954) Bukry, 1973	0.74	0.00	0.00	0.00	0.00	NP14-NP23
Helicosphaera bipuncta de Kaenel & Bergen, in Boesiger et al. 2017	0.93	0.00	0.00	2.00	0.91	NP22-NN4
Pontosphaera enormis (Locker, 1967) Perch-Nielsen, 1984	0.52	0.00	0.00	0.00	0.00	NP10-NP25
Pontosphaera latielliptica (Baldi-Beke, in Baldi-Beke & Baldi 1974) Perch-Nielsen 1984	1.47	0.00	0.00	1.00	1.82	NP10-NP24
*Reticulofenestra bisecta (*Hay, Mohler and Wade, 1966) Roth, 1970	0.00	0.00	0.85	0.00	0.00	NP17-NN1
Reticulofenestra daviesii (Haq, 1968) Haq, 1971	0.74	0.92	1.30	4.00	0.91	NP14-NN2
Reticulofenestra lockeri Müller, 1970	8.54	6.56	0.00	20.00	18.18	NP17-NN2
Reticulofenestra minuta Roth, 1970	5.88	2.36	0.00	2.00	0.00	NP13-NN16
Reticulofenestra ornata Müller 1970	65.44	52.46	38.71	20.00	40.91	NP22-NP24
Reticulofenestra umbilicus (Levin, 1965) Martini & Ritzkowski, 1968	0.00	0.00	0.00	0.00	1.82	NP16--NP22
Sphenolithus moriformis (Brönnimann & Stradner, 1960) Bramlette & Wilcoxo	0.00	0.00	0.00	0.00	0.91	NP5-NN15
Sphenolithus pseudoradians Bramlette & Wilcoxon, 1967	0.89	0.00	0.00	0.00	0.00	NP15-NP24
Syracosphaera sp.	0.00	0.00	0.00	5.00	0.91	
Thoracosphaera sp.	0.00	0.00	1.08	0.00	0.00	

Supplementary Table 1: Diversity, abundance and stratigraphic distribution of calcareous nannofossils recorded from *Necroteuthis*-bearing sediment.

locality	No. level	sample	$\delta^{13}C_{carb}$	$\delta^{18}O_{carb}$	StDev C	StDev O
Csillaghegy	**JS1**	2622.15	**-2.27**	**-5.87**	0.011	0.022
Csillaghegy	**JS2**	3031.32	**-2.46**	**-6.01**	0.010	0.014
Csillaghegy	**JS3**	2444.14	**-2.38**	**-5.39**	0.011	0.029
Csillaghegy	**JS4**	4228.73	**-2.41**	**-5.74**	0.016	0.020
Csillaghegy	**JS5**	2781.15	**-2.34**	**-5.64**	0.008	0.030
Csillaghegy	**JS6**	2394.42	**-2.50**	**-5.72**	0.006	0.010
Csillaghegy	**JS7**	1428.39	**-2.50**	**-5.56**	0.009	0.013
Csillaghegy	**JS8**	2325.24	**-2.49**	**-5.58**	0.013	0.035
Csillaghegy	**JS9**	4039.36	**-2.45**	**-5.15**	0.008	0.005
Csillaghegy	**JS10**	3751.30	**-2.45**	**-5.38**	0.011	0.013
Csillaghegy	**JS11**	3440.80	**-2.59**	**-5.57**	0.008	0.013
Csillaghegy	**JS12**	3051.95	**-2.40**	**-5.32**	0.017	0.011
Csillaghegy	**JS13**	2003.81	**-2.61**	**-6.13**	0.016	0.016
Csillaghegy	**JS14**	1846.05	**-3.25**	**-5.92**	0.020	0.025
Csillaghegy	**JS15**	1802.10	**-2.48**	**-5.39**	0.026	0.023
Csillaghegy	**JS16**	1891.25	**-2.45**	**-5.49**	0.032	0.036
Csillaghegy	**JS17**	3066.10	**-0.93**	**-2.73**	0.041	0.028
Csillaghegy	**JS18**	3190.09	**-0.98**	**-2.01**	0.022	0.039
Csillaghegy	**JS19**	3646.33	**-1.08**	**-1.99**	0.022	0.013

Supplementary Table 2: Table of measured stable isotopes $\delta^{13}C_{carb}$ and $\delta^{18}O_{carb}$ from the bulk rock of *Necroteuthis*-bearing specimen (blue – Tard Clay Fm.) and overlying Kiscell Clay Fm. yielding *Archaeosepia* cuttlebones (green). Level with gladius highlighted by grey.

No. analysis	No.	Peak Nr	Rt	Ampl 44	BGD 44	Area All	$\delta^{13}C/\delta^{12}C$	$\delta^{13}C_{org}$
82009	**JS1**	3	165.9	3841	10.9	71.768	**-26.7229**	**-26.75**
82010	**JS2**	3	165.1	3467	10.4	64.622	**-26.6731**	**-26.72**
82011	**JS3**	3	165.3	4211	10.6	78.555	**-26.8036**	**-26.80**
82012	**JS4**	3	165.1	3698	10.7	69.137	**-26.7638**	**-26.76**
82013	**JS5**	3	165.5	3303	10.6	62.035	**-26.8126**	**-26.81**
82014		3	165.3	3800	10.3	71.299	**-26.8724**	**-26.87**
82015	**JS7**	3	165.1	3406	10.7	63.637	**-26.9282**	**-26.93**
82016	**JS8**	3	165.1	3752	10.8	70.184	**-27.0627**	**-27.06**
82017	**JS9**	3	165.3	3500	10.2	65.3	**-26.6372**	**-26.64**
82018	**JS10**	3	165.3	3346	10.2	62.783	**-26.8007**	**-26.80**
82019	**JS11**	3	165.5	3264	10.0	61.167	**-26.7319**	**-26.73**
82020	**JS12**	3	164.7	3748	10.4	69.712	**-26.7389**	**-26.74**
82021	**JS13**	3	164.9	3274	10.4	60.656	**-26.7269**	**-26.73**
82022	**JS14**	3	165.5	3181	10.1	60.768	**-26.4558**	**-26.46**
82023	**JS15**	3	165.7	4118	10.2	77.684	**-27.0697**	**-27.07**
82024	**JS16**	3	164.9	3387	10.5	63.312	**-26.8644**	**-26.86**
82025	**JS1**	3	165.3	4188	10.3	78.645	**-26.7678**	control
82026	**JS2**	3	165.3	4046	10.0	75.666	**-26.7638**	control

Supplementary Table 3: Table of measured stable isotope $\delta^{13}C_{org}$ from the bulk rock of *Necroteuthis*-bearing specimen. Level with gladius highlighted by grey.
