# Supplementary material for: Fossil evidence for vampire squid inhabiting oxygen-depleted ocean zones since at least the Oligocene
Source: Commun Biol. 2021 Feb 18;4:216. doi: 10.1038/s42003-021-01714-0 (PMC7893013; doi:10.1038/s42003-021-01714-0)
Supplement: Supplementary file 3 — Reporting Summary [file 42003_2021_1714_MOESM3_ESM.pdf]

nature research

Corresponding author(s): Martin Košťák

Last updated by author(s): Dec 7, 2020

Reporting Summary

Nature Research wishes to improve the reproducibility of the work that we publish. This form provides structure for consistency and transparency in reporting. For further information on Nature Research policies, see our Editorial Policies and the Editorial Policy Checklist.

Statistics

For all statistical analyses, confirm that the following items are present in the figure legend, table legend, main text, or Methods section.

n/a	Confirmed	
☐	☒	The exact sample size (n) for each experimental group/condition, given as a discrete number and unit of measurement
☐	☒	A statement on whether measurements were taken from distinct samples or whether the same sample was measured repeatedly
☒	☐	The statistical test(s) used AND whether they are one- or two-sided<br>*Only common tests should be described solely by name; describe more complex techniques in the Methods section.*
☒	☐	A description of all covariates tested
☒	☐	A description of any assumptions or corrections, such as tests of normality and adjustment for multiple comparisons
☒	☐	A full description of the statistical parameters including central tendency (e.g. means) or other basic estimates (e.g. regression coefficient) AND variation (e.g. standard deviation) or associated estimates of uncertainty (e.g. confidence intervals)
☒	☐	For null hypothesis testing, the test statistic (e.g. F, t, r) with confidence intervals, effect sizes, degrees of freedom and P value noted<br>*Give P values as exact values whenever suitable.*
☒	☐	For Bayesian analysis, information on the choice of priors and Markov chain Monte Carlo settings
☒	☐	For hierarchical and complex designs, identification of the appropriate level for tests and full reporting of outcomes
☒	☐	Estimates of effect sizes (e.g. Cohen's d, Pearson's r), indicating how they were calculated

Our web collection on statistics for biologists contains articles on many of the points above.

Software and code

Policy information about availability of computer code

Data collection	no commercial or open source software was used
Data analysis	no commercial or open source software was used

For manuscripts utilizing custom algorithms or software that are central to the research but not yet described in published literature, software must be made available to editors and reviewers. We strongly encourage code deposition in a community repository (e.g. GitHub). See the Nature Research guidelines for submitting code & software for further information.

Data

Policy information about availability of data

All manuscripts must include a data availability statement. This statement should provide the following information, where applicable:

- Accession codes, unique identifiers, or web links for publicly available datasets
- A list of figures that have associated raw data
- A description of any restrictions on data availability

Provide your data availability statement here.

Field-specific reporting

Ecological, evolutionary & environmental sciences study design

All studies must disclose on these points even when the disclosure is negative.

Study description	Survivory strategy of deep sea vampyre squid based on fossil material from the Oligocene of Hungary.
Research sample	Necroteuthis hungarica - fossil
Sampling strategy	Samples were taken and drilled from the sediment surroundink the sample.
Data collection	M.K. and J.S. had inicial idea to describe the material, investigation, conceptualization, data interpretations. M.K., J.S., D.F., I.F., K.H., N.H., A.T., A.C., R.M., J.Š.: performed investigation, methodology, writing - original draft, writing - review & editing, with input of all authors. K.H. and N.H. micropalaeontological analysis. A.C., R.M., J.Š.: Geochemical analysis. M.M.: Visualization, SEM analysis. A.T. proof-read and corrected the entire text, data interpretations.
Timing and spatial scale	Continuously since 2018 to the beginning of 2020
Data exclusions	No data were excluded
Reproducibility	All attempts were succesful
Randomization	Not relevant - a single specimen has been studied
Blinding	Not relevant - see above

Did the study involve field work? ☐ Yes ☒ No

Reporting for specific materials, systems and methods

We require information from authors about some types of materials, experimental systems and methods used in many studies. Here, indicate whether each material, system or method listed is relevant to your study. If you are not sure if a list item applies to your research, read the appropriate section before selecting a response.

Materials & experimental systems

n/a	Involved in the study	
☒	☐	Antibodies
☒	☐	Eukaryotic cell lines
☐	☒	Palaeontology and archaeology
☒	☐	Animals and other organisms
☒	☐	Human research participants
☒	☐	Clinical data
☒	☐	Dual use research of concern

Methods

n/a	Involved in the study	
☒	☐	ChIP-seq
☒	☐	Flow cytometry
☒	☐	MRI-based neuroimaging

Palaeontology and Archaeology

Specimen provenance	Clay pit near Budapest ("Ziegelfabrik von Csillaghegy, NNW – Budapest, Kisceller Ton"). The locality, i.e. the Csillag-hegy Brickyard is also known as Péter-hegy (or Péterhegy). The clay-pit, was refilled and does not exist any more.
Specimen deposition	Specimen (holotype) housed under No. M59/4672 - Hungarian Natural History Museum
Dating methods	Micro CT – Micro-computed tomography (μ-CT) imaging was performed with phoenix v|tome|x L 240 device, developed by GE Sensing & Inspection Technologies. Investigated samples were analyzed by using 240 kV/320W microfocus tube. Scanning parameters were set as follows. For Necroteuthis sample: voltage 200 kV, current 250 μA, projections 2500, average 3, skip 1, timing 500 ms, voxel size 80 μm and 0,5 mm Cu filter. After the scanning process, 3D data sets were evaluated with VG Studio Max 2.2. For the address of storage space see link at Supplementary materials - Supplementary Micro CT imaging.<br>Fourier-transform Infrared spectroscopy (FTIR). The infrared spectra were recorded by micro-ATR technique on a Thermo Nicolet iN10 FTIR microscope using Ge crystal in the 675 – 4000 cm-1 region (2 cm-1 resolution, Norton–Beer strong apodization, MCT/A detector). Standard ATR correction (Thermo Nicolet Omnic 9.2 software) was applied to the recorded spectra. Several miniature samples (< 1 mm) of dark-coloured fossilized material were analyzed by infrared spectroscopy using a micro-ATR technique. Reference spectra for gypsum and (hydroxyl)apatite were taken from the RRUFF online database of spectroscopic and chemical data of minerals.<br>Stable isotope record. Stable C and O isotopes in carbonate fraction of clay sediments were analyzed on isotope-ratio mass spectrometer (IRMS) MAT253, coupled with Kiel IV device for semi-automated carbonate preparation (ThermoScientific). 40-100 micrograms of milled powder were loaded into borosilicate glass vials, evacuated and digested in anhydrous phosphoric acid at 70°C following method85. Yielded CO2 gas was cryogenically purified and introduced into the IRMS via dual-inlet interface. Isotope composition was measured against CO2 reference gas and raw values were calibrated using international reference material NBS18 and two working standards with δ13C = 5.014‰, +2.48‰, -9.30‰ and δ18O = -23.2‰, -2.40‰, -15.30‰, respectively. The values are reported as permil vs. PDB, precision of measurement is 0.02 ‰ for carbon and 0.04 ‰ for oxygen.<br>Stable carbon isotopes of organic matter were measured on mass spectrometer MAT253, coupled to elementar analyzer Flash2000 HT Plus (ThermoScientific). Residues after digestion in hydrochloric acid of about 900-1300 micrograms were wrapped into tin capsules and combusted in a stream of helium at 1000°C in quartz tube packed with tungsten oxide and electrolytic copper. Purified CO2 gas was separated from other gases on capillary GC column (Poraplot Q, Agilent) and introduced into IRMS in continuous flow mode. Raw isotope ratios measured against CO2 reference gas were calibrated to PDB scale using two international reference materials (USGS24 carbon, USGS41 glutamic acid) and two working standards, with δ13C values -16.05, +37.76, -39.79, -25.60‰ , respectively. All the values are reported in permil PDB, precision measured on standards is 0.11 permil. Standard deviation = 0.106 ‰.<br>SEM and imaging. The fossilized gladius was examined at the Institute of Geology and Palaeontology, Faculty of Science, Charles University in Prague by scanning electron microscope (SEM) JEOL-6380LV at 20, 25 and 30 kV and at 1.7–10 k X magnification. Microscopic gladius remains as well as the bulk rock fragment with Ca-nannofossils were coated with gold and investigated in the low and high vacuum modes. The macro-photo of Necroteuthis specimen was taken using the camera Canon EOS 600D. Photographs were improved using CorelDRAW X7 and Corel Photo-Paint X7 graphic softwares.<br>Microfossil investigation. Microfossils were collected and determined using Olympus SZ61 binocular stereoscopic microscope Olympus B750 and optical Zeiss Axiolab 5 microscope and documented by SEM microscope JEOL-6380LV. Determination of foraminifers is in accordance with published methodics. Paleoecological parameters were evaluated on the presence and dominance of taxa exhibiting special environmental significance. The Benthic Foraminifera Oxygen Index (BFOI)36 was determined to assess bottom-water concentrations of dissolved oxygen. The index is based on proportion of oxic, suboxic and dysoxic indicator species of benthic foraminifera. The Kaiho´s classification of benthic foraminifera to these three groups was used. Because at least one oxic species was found, we used the following equation for counting of the BFOI in agrement with published data:<br>{[O / (O + D)] ´ 100}<br>Where: O – proportion of oxic species, D – proportion of dysoxic species. Values of the BFOI index can vary from -100 to +100, higher the values indicate higher oxygen concentration.<br>Taphonomic analysis of foraminiferal assemblages was performed following the concept. Beside the foraminifers, the organic walled cysts and algae were counted in wash residuum. Calcareous nannoplankton was studied by both optic microscope (magnification 1000 x, crossed and parallel nicols) and scanning electron microscope (SEM). Slides for optic microscopy were prepared according to.

☒ Tick this box to confirm that the raw and calibrated dates are available in the paper or in Supplementary Information.

Ethics oversight	No ethical approval. Hungarian Natural History Museum is a participant of the research.

Note that full information on the approval of the study protocol must also be provided in the manuscript.
